# Yunmen (LU 2) combined with neck-seven-acupoint acupuncture for arm numbness caused by cervical spondylotic radiculopathy: A case report

**DOI:** 10.1097/MD.0000000000026151

**Published:** 2021-06-04

**Authors:** Yi-Mei Zhang, Xing-Jun Han, Yong-Cheng Wang, Hong-Ling Jia, Xiao-Fen Yuan, Xian-Liang Meng, Zhao-Yu Li, Guo-Feng Zhou

**Affiliations:** a Department of Acupuncture and Moxibustion; b Department of Prevention and Health Centres, Second Affiliated Hospital of Shandong University of Traditional Chinese Medicine; c Department of Cardiovascular, Affiliated Hospital of Shandong University of Traditional Chinese Medicine, Jinan; d Department of Cardiovascular, Taian Traditional Chinese Medicine Hospital, Taian; e Departments of First Clinical Medical, Shandong University of Traditional Chinese Medicine, Jinan, China.

**Keywords:** cervical spondylotic radiculopathy, neck-seven-acupoint, numbness, Yunmen acupoint

## Abstract

**Rationale:**

Cervical spondylotic radiculopathy (CSR) is a common sensory, motor, and reflex disorder. Numbness, a common subjective symptom of CSR, lacks objective quantitative indicators and recognized effective treatments, but is also difficult to recover from. We present a case report describing a traditional acupuncture treatment for CSR, utilizing a special acupuncture method and point, namely the Yunmen point.

**Patient concerns:**

A 40-year-old woman presented with unilateral arm numbness caused by CSR.

**Diagnoses:**

A diagnosis of CSR was made in the orthopedic department of a local hospital.

**Interventions:**

We attempted acupuncture at the Yunmen (LU 2) acupoint combined with neck-seven-acupoint under computed tomographic guidance.

**Outcomes:**

After 10 times treatment sessions, the patient no longer experienced weakness, coldness, or numbness in the affected upper limb. In addition, the stiffness in the neck and shoulders was reduced. On physical examination, the patient's left brachial plexus traction test was negative; reassessment of the CSR-20-point score scale showed a perfect score, and the visual analog scale score was 0.

**Lessons:**

Our report indicates that acupuncture at the LU 2 acupoint combined with neck-seven-acupoint is effective in treating numbness and coldness of the arm, and other neurological symptoms caused by cervical spondylosis. Moreover, with the appropriate acupuncture technique, the risk of acupuncture at the LU 2 acupoint can be minimized.

## 1 Introduction

Cervical spondylotic radiculopathy (CSR) is a degenerative disorder characterized by numbness and is one of the main clinical symptoms. At present, there are relatively few clinical studies on CSR numbness, with a lack of systematic and effective therapies.^[1–3]^ Clinical trials have shown that acupuncture has the same analgesic effect as drug therapy and is safe and easily accepted. Moreover, the National Institute for Health and Care Excellence clinical guidelines explicitly recommend acupuncture for CSR.^[4]^ However, there is no clear report on whether acupuncture plays an effective role in nerve compression symptomatic treatment, such as upper limb numbness in CSR patients. Our research showed that the patient who had obvious numbness of the upper limbs felt the relief of nerve compression symptoms after acupuncture at the Yunmen (LU 2) acupoint combined with neck-seven-acupoint.

## 2 Case report

A 43-year-old woman visited our department on July 7, 2020, with a chief complaint of numbness, weakness, and coldness of the left upper extremity, with radial pain from the shoulder to the elbow. She had been diagnosed with CSR 2 years ago and was treated with neurotrophic drugs and painkillers, including MeCobalamin tablets (0.5 mg, PO, TID) and loxoprofen sodium tablets (60 mg, PO, TID). However, as the symptoms were not completely relieved after intermittent treatment for 3 months, the patient sought acupuncture. On physical examination, the left brachial plexus traction test was positive, and the results of the thoracic outlet syndrome, cubital tunnel syndrome, and carpal tunnel syndrome tests were all negative, bilateral Hoffman sign was not elicited, upper limb muscle strength and tension were normal, and the patient's numbness and pain were scored as 10 and 6 using Tanaka Jingjui's CSR-20-point score and the visual analog scale (VAS), respectively.^[5,6]^

**Figure 1:**
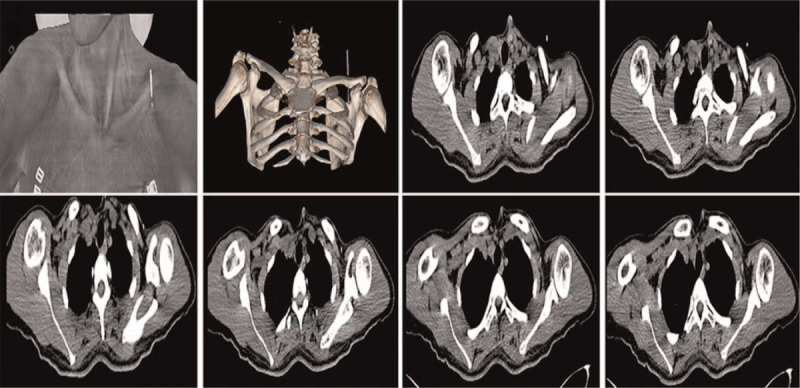
Position relationship between acupuncture points and pleural cavity on computed tomography.

Acupuncture methods: (1) Acupoint selection: The LU 2 acupoint was selected for the treatment. The patient was instructed to lie down with her hands akimbo and elbows leaning forward slightly to fully separate the thorax and shoulder joint, fully expose the acupoint, and form a triangular depression visible at the lower edge of the clavicle's outer end. The depression on the inner edge of the scapula's coracoid process is the LU 2 acupoint. It is recorded in the A-B Classic of Acupuncture and Moxibustion that improper acupuncture at the LU 2 acupoint will lead to pneumothorax, which is why this is seldom used clinically, except in the treatment of cough. To ensure the patient's physical safety, the operation at the LU 2 acupoint was performed under computed tomography (CT) guidance, as shown in Figure 1. (2) Acupuncture operation: First, the skin over the LU 2 acupoint was disinfected with disposable 75% alcohol swabs. With the patient lying down as previously mentioned, a sterile needle (Huatuo, Medical Supplies Factory Co., Ltd., Suzhou, China; sterile needle length, 40 mm; diameter, 0.30 mm) was inserted into the acupoint at an approximate depth of 1 cun (1 cun = 25 mm). Then, the inserted needle was gently lifted to the subcutaneous tissue, turned back vertically as above, and finally rotated in a small range of the acupoint. The 2 operations were repeated alternately for 1 min until the patient experienced a special feeling, such as local acid swelling, ant bite pain, and other special feelings. After stimulation, the needle remained in the acupoint for 20 min before being pulled out.

**Figure 2:**
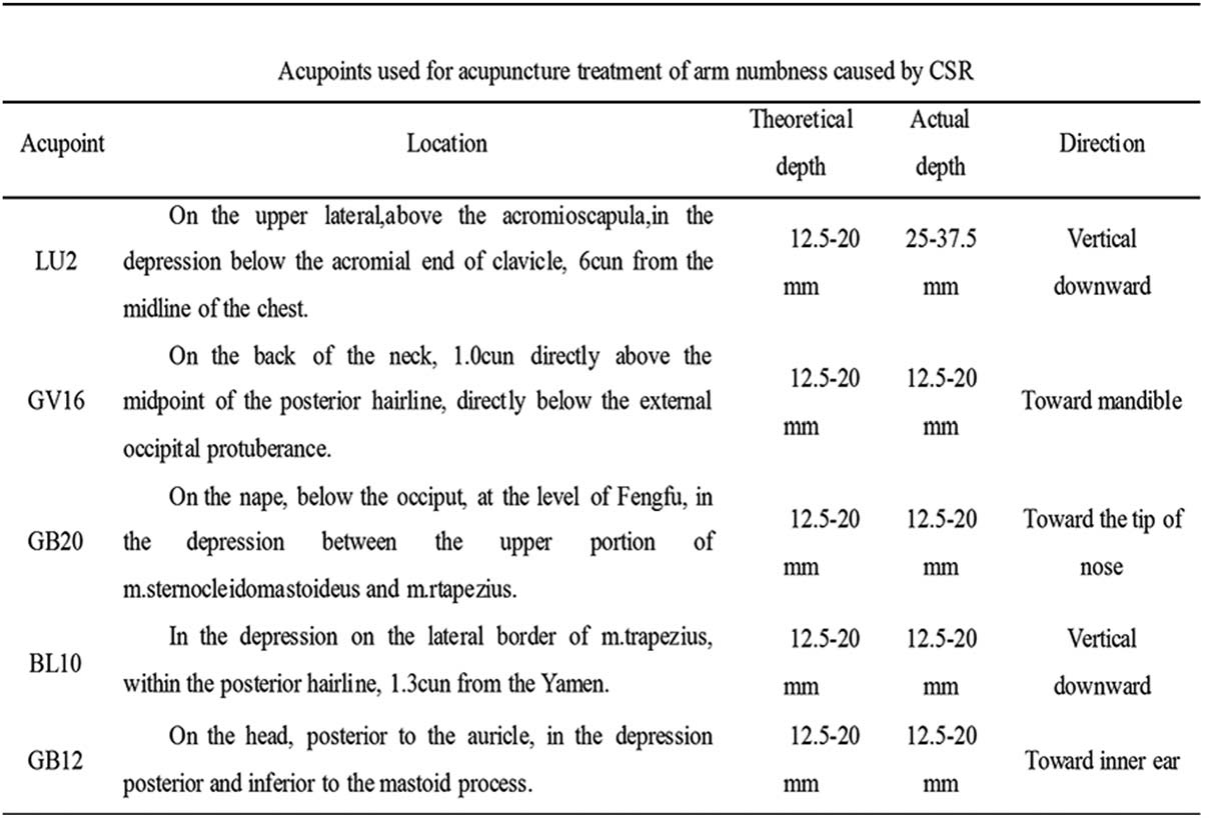
Acupoints for acupuncture treatment of arm numbness caused by CSR. CSR = cervical spondylotic radiculopathy.

After the LU 2 acupoint operation, the patient was instructed to lie in the right lateral decubitus position and was treated with the neck-seven-acupoint, including Fengfu (GV 16), Fengchi (GB 20), Tianzhu (BL 10), and Wangu (GB 12). Apart from the GV 16 acupoint, the other 6 acupoints were bilaterally symmetric. Similar to the LU 2 acupoint stimulation in which the needling sensation of the neck-seven-acupoint was elicited, these needles were also retained for 20 minutes, with manipulation once every 10 minutes, before removal. The specific depth and direction of the acupoints are shown in Figure 2.

After all the needles were pulled out at the end of the first treatment session, the patient described alleviation of numbness and a feeling of relaxation of her left arm and shoulder, although coldness was still not completely relieved. The patient was advised to undergo treatment on alternate days and 5 times during the course of treatment. On July 15, 2020, the patient received a fifth acupuncture treatment. Evaluation of treatment results showed that pain and discomfort of the neck and shoulder were relieved, although numbness and coldness of the upper limb skin persisted, but to some extent, symptomatic relief. She was assessed for her CSR-20-points score, which ranged from 10 to 16 after her fifth treatment session. Following this remedy pattern, the patient underwent 5 more treatment sessions; the last treatment session was on July 25, 2020. At the end of the treatment, the patient no longer experienced weakness, coldness, or numbness in the left upper limb. In addition, the stiffness in the neck and shoulders was reduced. On physical examination, the patient's left brachial plexus traction test was negative, reassessment of the CSR-20-point score scale showed a perfect score, and the VAS score was 0. No recurrences occurred during follow-up in the next 2 months. Written informed consent was obtained from the patient for publication of this case report and accompanying images, and all procedures involving the patient were conducted in accordance with the ethical standards of the Ethics Committee of the Second Affiliated Hospital of Shandong University of Chinese Medicine.

## 3 Discussion

CSR is a disease that involves ischemia of compressed cervical nerve roots.^[7]^ It is widely known that conservative therapies can relieve CSR symptoms, although pharmacotherapy as a part of a multimodal and initial approach can play a clinical role with its effect not being all that ideal.^[8,9]^ Traditional Chinese medicine interventions have been widely used for the management of CSR, and acupuncture, as a non-invasive treatment, is one of the most popular because many clinicians have reported their experiences of its effects on the radicular syndrome, such as pain and paresthesia.^[10–12]^ To the best of our knowledge, there is no related research on acupuncture treatment at the LU 2 acupoint for numbness, which our study found to be effective for numbness caused by CSR.

In this case, we report the case of a 40-year-old woman with arm numbness caused by CSR and received acupuncture therapy. We guided the patient to select a certain posture and adopted specific techniques to create a stimulation sensation. To ensure and further verify the safety of the acupuncture method, we chose to conduct it under CT guidance. Herein, the operation method of the LU 2 acupoint was quite different from the traditional acupuncture routine. The routine acupuncture method of the LU 2 acupoint involved obliquely inserting 0.5–0.8 Â toward the lateral aspect of the thorax instead of the inside, with the intention of avoiding injuring the lungs. Our clinical observation showed that the efficacy of directly needling 1–1.5 cun into the LU 2 acupoint was much better than conventional acupuncture, and it was more secure and effective because pleural and lung damage with concomitant pneumothorax was avoided. Clinical studies have confirmed that acupuncture can enhance endogenous neurogenesis and ameliorate neurological impairments.^[13,14]^ Moreover, acupuncture can accelerate local blood circulation and restore hemodynamic state, following the principle that the greater the insertion depth and the greater the stimulation induced by the needle, the greater its effects within certain limits.^[15,16]^ Similar to previous studies, we speculated that this may be due to the insertion depth, which more likely stimulated the oppressed nerves and improved the state of nervous ischemia, leading to the improvement of neurological symptoms.

Acupuncture of the LU 2 acupoint has a good therapeutic effect on the numbness of the upper limbs caused by CSR since the key problem of numbness is cervical disc degeneration. Studies have also shown that neck-seven-acupoint acupuncture can improve vertigo symptoms caused by the vertebral artery type of cervical spondylosis through blood supply improvement.^[15,17]^ Additionally, acupuncture at these acupoints can also regulate Dll 4 and Hes 1 mRNA and protein expression in the intervertebral disc tissue to defer degeneration.^[18]^ According to the patient's description of the affected area of the left upper limb, it was likely to be related to the compression of the C5 nerve root. Inside the neck-seven-acupoint, the C3–C5 spinal nerve is spread as the needles are inserted into these acupoints to stimulate the posterior ramus of the spinal nerve, which is the main nerve supply. This may induce reflex effects throughout the spinal segment and alleviate radicular symptoms in the upper extremities.^[2]^ It has been proven that acupuncture at the cervical acupoints can relieve the oppressed sympathetic nerves of the neck and reduce the excitability of the sympathetic nerve to relieve symptoms of numbness and pain caused by cervical compression.^[15,19]^

Direct needling of the LU 2 acupoint has a good therapeutic effect on the numbness of the upper limbs caused by CSR; when combined with the neck, this effect can be enhanced in the treatment of cold skin, limb paresthesia, and other neurological CSR symptoms. To verify the effectiveness of acupuncture treatment, further studies with sufficient sample sizes are needed to provide evidence-based proof and explore the underlying mechanisms of action of this treatment.

## Author contributions

**Conceptualization:** Yi-Mei Zhang, Xing-Jun Han, Yong-Cheng Wang.

**Data curation:** Yi-Mei Zhang, Xing-Jun Han, Yong-Cheng Wang.

**Formal analysis:** Yi-Mei Zhang, Xing-Jun Han, Yong-Cheng Wang.

**Funding acquisition:** Yi-Mei Zhang.

**Investigation:** Yi-Mei Zhang, Xing-Jun Han, Yong-Cheng Wang, Hong-Ling Jia, Xiao-Fen Yuan.

**Methodology:** Yi-Mei Zhang, Yong-Cheng Wang, Hong-Ling Jia, Xiao-Fen Yuan, Zhao-Yu Li, Guo-Feng Zhou, Xing-Jun Han.

**Project administration:** Yi-Mei Zhang, Yong-Cheng Wang.

**Resources:** Yi-Mei Zhang, Yong-Cheng Wang.

**Software:** Yi-Mei Zhang, Yong-Cheng Wang, Hong-Ling Jia, Xiao-Fen Yuan, Xian-Liang Meng, Zhao-Yu Li, Guo-Feng Zhou.

**Supervision:** Yi-Mei Zhang, Yong-Cheng Wang, Hong-Ling Jia, Xiao-Fen Yuan, Xian-Liang Meng, Zhao-Yu Li, Guo-Feng Zhou, Xing-Jun Han.

**Validation:** Yi-Mei Zhang, Yong-Cheng Wang, Hong-Ling Jia, Xiao-Fen Yuan, Xian-Liang Meng, Guo-Feng Zhou.

**Visualization:** Yi-Mei Zhang, Yong-Cheng Wang, Xiao-Fen Yuan, Zhao-Yu Li, Xian-Liang Meng.

**Writing – original draft:** Yi-Mei Zhang, Yong-Cheng Wang, Xing-Jun Han.

**Writing – review & editing:** Yi-Mei Zhang, Yong-Cheng Wang, Xing-Jun Han.
